# Recent Progress of Biomaterials-Based Epidermal Electronics for Healthcare Monitoring and Human–Machine Interaction

**DOI:** 10.3390/bios13030393

**Published:** 2023-03-17

**Authors:** Ningning Han, Xin Yao, Yifan Wang, Wenhao Huang, Mengjuan Niu, Pengcheng Zhu, Yanchao Mao

**Affiliations:** Key Laboratory of Materials Physics, Ministry of Education, School of Physics and Microelectronics, Zhengzhou University, Zhengzhou 450001, China

**Keywords:** biomaterials, epidermal electronics, healthcare monitoring, human–machine interaction

## Abstract

Epidermal electronics offer an important platform for various on-skin applications including electrophysiological signals monitoring and human–machine interactions (HMI), due to their unique advantages of intrinsic softness and conformal interfaces with skin. The widely used nondegradable synthetic materials may produce massive electronic waste to the ecosystem and bring safety issues to human skin. However, biomaterials extracted from nature are promising to act as a substitute material for the construction of epidermal electronics, owing to their diverse characteristics of biocompatibility, biodegradability, sustainability, low cost and natural abundance. Therefore, the development of natural biomaterials holds great prospects for advancement of high-performance sustainable epidermal electronics. Here, we review the recent development on different types of biomaterials including proteins and polysaccharides for multifunctional epidermal electronics. Subsequently, the applications of biomaterials-based epidermal electronics in electrophysiological monitoring and HMI are discussed, respectively. Finally, the development situation and future prospects of biomaterials-based epidermal electronics are summarized. We expect that this review can provide some inspirations for the development of future, sustainable, biomaterials-based epidermal electronics.

## 1. Introduction

With the rapid development of electronic devices regarding on-skin applications such as healthcare monitoring and human–machine interactions, traditional rigid electronic devices are facing challenges to meet the specific requirements of flexibility, stability, biocompatibility, etc. [1,2,3,4]. Recently, epidermal electronics has received increasing research attention due to its advantages of intrinsic softness and long-term wearable comfort for on-skin applications [5,6,7,8]. To be specific, compared with rigid electronic devices, epidermal electronics that mimic various properties of human skin can conformally attach onto human skin with irregular surfaces, which is favorable for reducing contact impedance and improving the fidelity of acquired electrophysiological signals [9,10,11,12,13]. More importantly, epidermal electronics can keep its mechanical and electrical properties stable even under severe stretching or twisting, which further makes it suitable for application fields including healthcare monitoring and HMI [14,15,16,17].

Although the rapid development of epidermal electronics has brought great benefits to daily life, the existing epidermal electronics mainly composed of synthetic materials are usually hard to degrade and may produce a large amount of electronic waste (e-waste) to the ecosystem, causing a series of environmental problems [18,19,20]. Furthermore, attaching these synthetic epidermal electronics onto human skin may cause serious adverse reactions such as allergy and stimulation, which brings along possible safety problems [21,22,23,24]. Besides synthetic materials, some inorganic materials, which are designed with some specific structures, such as serpentine structure [25,26], island-bridge structure [27,28] and Kirigami structure [29], can also be used to construct epidermal electronics. These types of epidermal electronics not only possess outstanding electrical performance, but are endowed with good stretchability by unique structures [30]. However, complex structural design adds cost for manufacture, and sophisticated preparation technology is also needed, which accordingly limits large-scale processing [31]. Therefore, developing new materials that possess the biodegradable and biocompatible properties is urgently needed to resolve these issues. Additionally, there are already some reports on constructions of epidermal electronics using some biocompatible and environmental-friendly polymers, which partly alleviates the mentioned problems [32,33]. However, obtaining these materials typically requires high-cost and time-consuming synthesis processes, hindering further development of sustainable and biocompatible epidermal electronics [34,35,36].

In comparison to synthetic polymer materials, biomaterials originally obtained from nature are emerging as promising candidates for the construction of next-generation epidermal electronics because of their unique merits [37,38,39]. In general, biomaterials are usually extracted from various living creatures including organisms, animals, and plants [40,41,42,43]. Besides the intrinsic advantages of biocompatibility and full biodegradability, biomaterials also have other appealing merits such as low cost to acquire, natural abundance and easier processing procedures [44,45]. To date, many reports have proposed epidermal electronics constructed by using various kinds of biomaterials including polysaccharides and proteins [46,47,48]. Additionally, their applications have proven great potential as the next-generation environmentally friendly wearable electronics [49,50,51]. In addition, biomaterials-based electronic devices can also be widely used in multiple on-skin applications, including but not limited to human–machine interactions (HMI), electrophysiological signals monitoring [52,53,54,55,56], biomedical implants and environmental monitoring [57].

In this review, we discuss recent research progress about biomaterials-based epidermal electronics in three aspects. In Section 2, the main biomaterials used to construct multifunctional epidermal electronics are reviewed, including proteins such as silk protein, gelatin, gluten and polysaccharides such as cellulose, starch and chitosan. In Section 3, we summarize the applications of biomaterials-based epidermal electronics in electrophysiological signals monitoring, including electrocardiograph (ECG), electromyography (EMG) and electroencephalogram (EEG). Then, we review the applications of biomaterials-based epidermal electronics in human–machine interactions such as robotic control, personal devices control and virtual reality (VR). In the final section, according to current development of biomaterials-based epidermal electronics, we prospect the future development direction for better practical applications. We expect that this review can offer inspiration to further innovation of high-performance biomaterials-based epidermal electronics.

## 2. Biomaterials-Based Epidermal Electronics

Compared to traditional electronic devices, biomaterials-based epidermal electronics possess the advantages of biodegradability, easy obtainment, environmental friendliness, excellent biocompatibility and low cost, which have gained increasing research attention in recent years [57,58,59,60]. To date, biomaterials-based epidermal electronics are usually prepared using two major categories of materials, including protein and polysaccharide [61,62,63]. Moreover, other biomaterials, for example lignin and shellac, can also be used to prepare epidermal electronics [61]. However, there is relatively less research on lignin and shellac, so we mainly focus on the recent research progress of biomaterial use in the epidermal electronics field, especially on silk protein, gelatin, gluten, cellulose, starch and chitosan (CS) in this section.

### 2.1. Protein Based Epidermal Electronics

#### 2.1.1. Silk Protein

**Figure 1:**
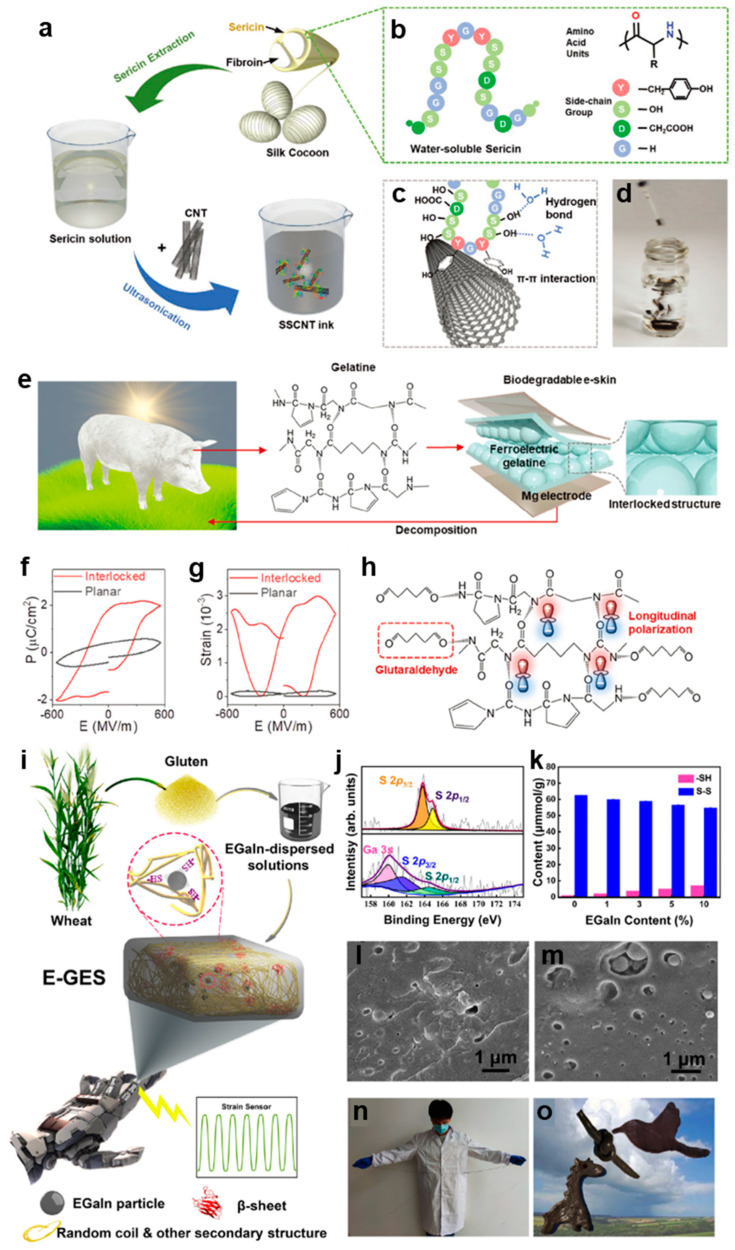
Fabrication processes and characterization of diverse protein-based epidermal electronics. (**a**) Schematic for extraction of sericin protein from silk cotton and preparation of SSCNT ink. (**b**) Chemical structure of sericin protein. (**c**) Schematic illustration showing π–π interaction between CNT and sericin, and hydrogen bond between water molecule and sericin. (**d**) Photograph of the SSCNT ink dispersing quickly in water [66]. (**e**) Structure and bio-decomposition of gelatin-based ferroelectric skin. (**f**) P-E hysteresis loop and (**g**) Strain hysteresis loop of interlocked and planar structured gelatin-based epidermal electronics. (**h**) Ferroelectric enhancement mechanism by the contribution of glutaraldehyde [73]. (**i**) Preparation process of the EGaIn/gluten-based e-skin (E-GES). (**j**) XPS spectra of E-GES without EGaIn (top) and with 5% EGaIn (bottom). (**k**) Changes of -SH and S-S content with the increasing of EGaIn content. (**l**,**m**) SEM images of E-GES without EGaIn and with 5% EGaIn. (**n**) Photograph of the stretching E-GES. (**o**) Photograph of E-GES with different shapes [77].

Silk protein, primarily composed of two components: silk fibroin and silk sericin, is one of the first utilized animal fiber proteins, in which silk fibroin approximately takes up more than 70% of the overall weight [64]. Recently, silk protein has been a promising biomaterial for epidermal electronics, mainly owing to its advantages of decent tensile resistance, toughness, biological compatibility and biodegradability [65]. Based on the considerable merits of silk protein, Liang et al. developed a biocompatible silk sericin–carbon nanotube hybrid ink (SSCNT) which could maintain electrical stability for up to a number of months [66]. The fabrication procedure of the SSCNT is shown in Figure 1a, in which sericin was extracted from a silk cocoon and dissolved in water to form sericin solution; then, CNTs were added to the solution with ultrasonication to obtain SSCNT ink. The chemical structure of water-soluble sericin is shown in Figure 1b, where amino acid units were bonded with diverse side-chain groups, including hydroxy and carboxymethyl. After mixing sericin with CNTs, π–π interactions were formed between aromatic amino acid residues of sericin and the surface of CNTs (Figure 1c). As a result, CNTs were uniformly dispersed in water to obtain a stable and biocompatible CNTs ink (Figure 1d) with the existence of silk sericin. Finally, as a sustainable biomaterial, the sericin modified-CNTs ink can be printed to obtain epidermal electronics for smart wearables.

#### 2.1.2. Gelatin

Apart from silk protein, gelatin, mainly composed of collagen [67,68,69], is also considered to be an ideal material candidate for epidermal electronics owing to its environmental abundance and biocompatibility [70]. Currently, the massive usage of personal electronic devices has produced a great amount of e-waste, causing serious environmental problems [71,72]. Hence, it is highly desirable to develop electronic devices that are made out of eco-friendly materials. To overcome the severe challenge of e-waste, Ko et al. demonstrated a kind of fully biodegradable ferroelectric epidermal electronics based on porcine skin gelatin that possesses the diverse advantages of biodegradability, flexibility and suitable pyro/piezoelectric coefficients [73]. Combing with the interlocked structure which widely exists in biological epidermal-dermal layers, gelatin-based epidermal electronics was able to precisely detect external pressure and temperature signals. Figure 1e illustrates the basic composition of the ferroelectric gelatin e-skin which can fully degrade without any e-waste footprint. In addition, zero noxious solvents were used when the sustainable epidermal electronics was prepared, which further proved its decent eco-friendliness. The ferroelectricity of the gelatin-based epidermal electronics was investigated by using the electric field (*E*)-induced hysteresis loop (Figure 1f). It can be seen that the interlocked structure possessed a better switchable polarization loop. Additionally, the strain hysteresis loop was also measured as shown in Figure 1g, which shows that the interlocked gelatin film has a higher electrostriction coefficient. To further enhance the ferroelectricity of gelatin film, glutaraldehyde was introduced to cross-link with gelatin, which made the structure of epidermal electronics anisotropic, as shown in Figure 1h. Based on the above results, the reported gelatin epidermal electronics could imitate the structure and functionality of human skin to simultaneously monitor and distinguish external pressure and temperature signals.

#### 2.1.3. Gluten Protein

Generally, protein-based epidermal electronics are designed following a sophisticated procedure, which makes it difficult to realize its capability of self-healing and tunable flexibility [74,75]. Compared with the above two proteins, gluten protein possesses a diversified dynamic chemical bond in addition to its biocompatibility, low-cost and sustainability, which could easily achieve self-healing and tunable flexibility [76]. Based on the merits, Chen et al. developed a gluten protein-based epidermal electronics that had a hybrid network by importing a cross linking agent: eutectic gallium indium alloy [77]. Figure 1i shows the fabrication process of the EGaIn/gluten-based e-skin (E-GES), in which the introduction of EGaIn could form metal coordination interaction with the free sulfhydryl (-SH) groups within gluten. Combining with another cross-linker, β-sheets, the E-GES was endowed with desired mechanical and self-healing ability. Figure 1j,k verify that EGaIn could bond with -SH, and the crosslinking extent increased with an increasing content of EGaIn. Additionally, the slight reduction of the disulfide bond has no negative influence on the structural integrity of E-GES, as shown in Figure 1l,m. Finally, the obtained E-GES could be stretched and shaped arbitrarily (Figure 1n,o), and act as epidermal electronics for human motion monitoring.

### 2.2. Polysaccharide Based Epidermal Electronics

#### 2.2.1. Cellulose

**Figure 2:**
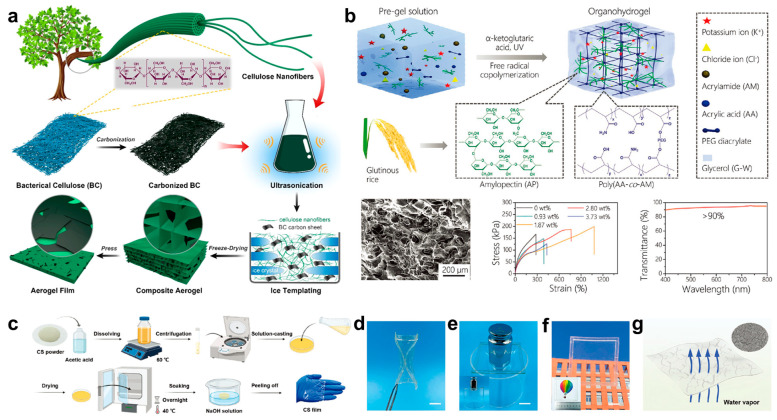
Fabrication processes and characterization of diverse polysaccharide-based epidermal electronics. (**a**) The preparation process of nanocellulose-based aerogel film [83]. (**b**) Schematic illustration showing the fabrication process and characteristics of amylopectin (AP) based hydrogel, including SEM image, strain-stress curves with different AP contents and transparency [93]. (**c**) Fabrication process of chitosan (CS) membrane. (**d**) Photograph showing flexibility of CS membrane. (**e**) Photograph of CS membrane pressed by a 500 g weight. (**f**) Outdoor and indoor (inset) images illustrating the transparency of CS membrane. (**g**) Image showing the breathability of CS membrane and SEM image (inset) of CS surface [98].

Cellulose, a kind of polysaccharide, is also a promising biomaterial for epidermal electronics applications. It is usually acquired from natural cotton, wood or bacteria [78,79,80]. In specific, bacterial nanocellulose (BC) extracted from bacteria has been regarded as an ideal candidate for environment-friendly epidermal electronics [81,82]. Recently, Chen et al. proposed a carbonized bacterial nanocellulose/cellulose nanofibrils (CBC/CNF)-based aerogel film [83]. Based on the aerogel film, a pressure sensor with advantages of fast response, dependability and good sensitivity was developed. Figure 2a schematically illustrates a detailed fabrication process of the aerogel film. First, multiple heating and cooling treatments were applied on BC to obtain carbonized BC; it was subsequently blended with CNF solution using sonication. Then, the mixed solution was poured into a petri dish, and then it was frozen by liquid nitrogen through a directional way. Additionally, the frozen CBC and CNF hybrid was freeze-dried and applied with a compression of 1 MPa to obtain the aerogel film. Ultimately, based on the pressure-dependent interaction between CBC/CNF-based composite aerogel and electrodes, a pressure sensor with rapid response and large-scale pressure detecting ability was developed, which was successfully used for communicating with smartphones.

#### 2.2.2. Starch

Starch, an edible and renewable biomaterial in nature, can be classified into two types in terms of chemical structure: amylose and amylopectin (AP) [84,85,86]. It can be easily extracted from various crops, including glutinous rice, corn and potato [87,88,89,90]. In starch, AP is a promising biomaterial for application in adhesive epidermal electronics owing to its intrinsic adhesive property, which originates from its rich branched structures that expose a large number of hydroxyl adhesive groups [91,92]. Zhou et al. developed an adhesive organohydrogel based on AP with desired adhesiveness, transparency, flexibility and conductivity [93]. Figure 2b shows the fabrication process of the adhesive epidermal electronics. Firstly, a thermal treatment was applied to AP solution to form AP gel, in which more hydroxyl groups were exposed. Then, AP gel was mixed with acrylic acid (AA), acrylamide (AM), chloride, glycerol and crosslinker poly (ethylene glycol) (PEG). Afterwards, α-ketoglutaric was added to the mixture and then treated with ultraviolet (UV) light. During the UV irradiation, copolymerization happened between AA and AM to form a poly (AA-co-AM) network, followed by crosslinking with the assistance of PEG to obtain excellent stretchability. Because of the existence of ion and glycerol, the obtained organohydrogel simultaneously possesses excellent conductivity and low temperature tolerance. In addition, it also has excellent elongation and transparency that could reach 1089% and 90%, respectively. Ultimately, the AP based hydrogel is able to act as an adhesive flexible epidermal electronics electrode for energy storage and human–machine interaction.

#### 2.2.3. Chitosan

Besides the above two polysaccharides, chitosan (CS), which can be extracted from chitin, is another excellent biomaterial because it has the advantages of being biocompatible, anticancer, bacteriostatic, abundant and biodegradable [94]. Hence, the CS can be widely used for epidermal electronics, medicine, chemical industry and biology [95,96,97]. Peng et al. reported a multi-functional chitosan based epidermal electronics which could provide more possibilities for future flexible devices [98]. Figure 2c shows the corresponding fabrication process of the epidermal electronics. First, CS powder and glycerol were blended with acetic acid solution and stirring was continued for several hours to obtain a yellow viscous solution. After being centrifuged at 10,000 rpm, the particle-free solution was casted and dried overnight to obtain a membrane. After it was soaked into NaOH solution and washed in distilled water, a CS membrane was obtained successfully after drying at room temperature. Figure 2d–f show that the obtained CS membrane has excellent flexibility, decent stress tolerance and high transparency, respectively. Figure 2g further shows that the CS membrane could also realize the capability of moisture permeability due to the micro-crack structure in the membrane. Finally, a gold nanofibers-based CS membrane was constructed as epidermal electronics for sensing pressure variation with quick response (70 ms) and a wide pressure detecting range (0–70 kPa).

In addition to proteins and polysaccharide, lignin and shellac are also widely used in epidermal electronics [99]. Typically, lignin, which is an abundant bio-polymer in plants, could endow epidermal electronics with excellent adhesion performance due to the abundant catechol group [100]. Moreover, there are many functional groups within lignin, such as a fatty hydroxyl group, phenolic hydroxyl group and carboxyl group, which could provide more possibilities for chemical modification and grafting copolymerization for lignin, resulting in the improvement of various properties, including conductivity, sensing property and absorbability [101]. Shellac, which usually finds its applications in nail polish, edible coatings in food and pharmaceutical industries, could also be applied in epidermal electronics [102]. As a water-insoluble, biodegradable, flexible and renewable biomaterial, shellac can act as a binder to connect conductive particles, which could obtain epidermal electronics with excellent waterproof properties [103].

## 3. Applications

### 3.1. Health Monitoring

The harmless, real-time and accurate detection of human electrophysiological signals is of great importance for judging human health condition [104,105,106]. Additionally, epidermal electronics that can provide seamless interaction with human skin plays an important role in acquiring these electrophysiological signals [107,108,109]. Among various materials for epidermal electronics, biomaterials have no harm to humans compared with other synthetic polymer-based epidermal electronics materials, owing to their good biocompatibility [110,111,112]. Hence, they are better candidates for epidermal electronics in electrophysiological signals acquisitions. In this section, the biomaterials-based epidermal electronics for recording multiple electrophysiological signals are reviewed, including ECG, EMG and EEG.

#### 3.1.1. ECG

**Figure 3:**
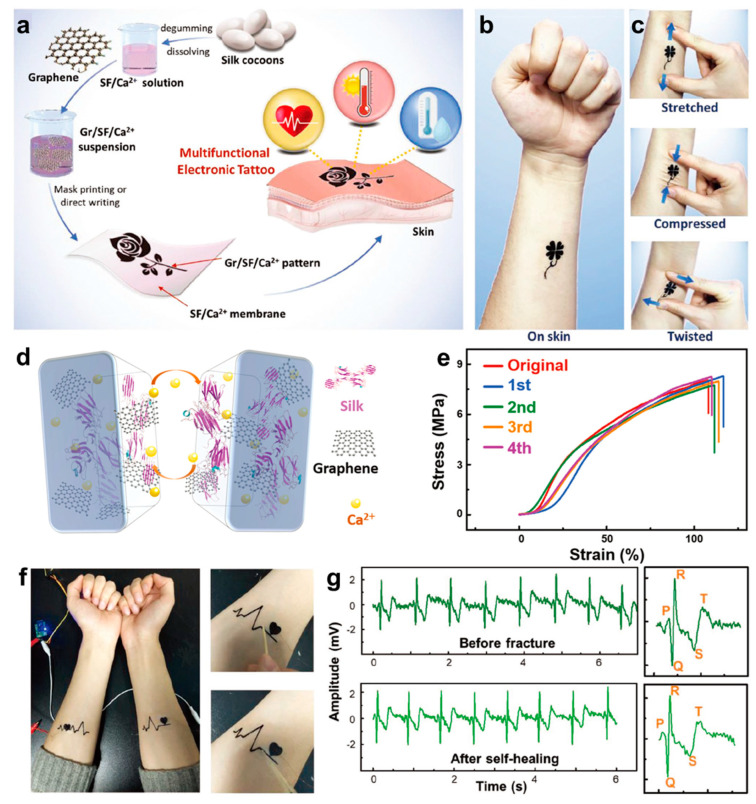
Preparation, characterization and application illustration of Gr/SF/Ca^2+^ E-tattoo. (**a**) Schematic procedures for synthesizing Gr/SF/Ca^2+^ multifunctional E-tattoo. (**b**) Photograph showing conformal contact between E-tattoo and human forearm. (**c**) Photograph showing the flexibility of E-tattoo on skin under stretching, compressing and twisting. (**d**) Schematic diagram of self-healing property of Gr/SF/Ca^2+^ E-tattoo. (**e**) Strain–stress curves of Gr/SF/Ca^2+^ E-tattoo after 0, 1, 2, 3 and 4 times of healing. (**f**) Photograph of Gr/SF/Ca^2+^ E-tattoo attached on forearms for ECG monitoring. The images on the right illustrate the fractured E-tattoo and recovered E-tattoo. (**g**) ECG signals acquired by original E-tattoo (top) and self-healed E-tattoo (bottom) [118].

ECG, one of the most frequently used inspections in clinic, can provide rich heart condition information for diagnosing a variety of heart diseases, including but not limited to arrhythmia, cardiac hypertrophy and myocardial ischemia [2,113,114,115]. Electronic tattoos (E-tattoos) which are mainly constituted by biomaterials have good potential to monitor ECG signals through noninvasive and biocompatible methods [116,117]. In addition, they can realize conformal contact with the microscopic morphology of human skin, which guarantees the acquisition of a more stable ECG signal. Thereby, based on graphene, Ca^2+^ and silk fibroin (SF), Wang et al. developed a kind of multifunctional Gr/SF/Ca^2+^ E-tattoo that is capable of monitoring ECG signals [118]. Figure 3a presents the detailed fabrication process of the E-tattoo. First, degummed SF extracted from silk cocoons was blended with CaCl_2_/formic acid solution (weight ratio of 1:20) and was kept stirring to obtain a SF/Ca^2+^ solution. Then graphene was added into SF/Ca^2+^ solution and treated by ultrasonication. The obtained suspension was then directly written, or screen printed onto a SF/Ca^2+^ film. Thus, a multifunctional E-tattoo for ECG detection, temperature and humidity sensing was obtained. For attaching the E-tattoo on human skin, a drop of water was coated on the target skin in order to make SF/Ca^2+^ film contact conformally with skin, and then the E-tattoo was placed on human skin to serve as an electrode for ECG monitoring. As shown in Figure 3b, conformal contact between E-tattoo and human skin could be observed. Figure 3c shows that after being stretched, compressed and twisted, the E-tattoo could still keep reliable adhesion with the human arm without damage or delamination. In addition to damage resistance, the E-tattoo also possesses excellent self-healing ability comparable to that of human skin, and the principle of the self-healing property is illustrated in Figure 3d. When the SF-based E-tattoo was broken into two pieces, they could be rejoined together as a whole only by adding a certain amount of water to the damaged area. This was because swelling would happen between SF chains if water was added, which led to physical contact between the two separate parts. Further, reversible hydrogen and coordination bonds within silk, graphene and Ca^2+^ would interconnect dynamically to achieve the self-healing property. Figure 3e shows that after four cycles of the cutting and healing, the tensile stress–strain curves of the E-tattoo only showed slight degradation.

Based on the above characteristics, the ECG signal was recorded by two E-tattoos attached to both arms (Figure 3f), which had a low contact impedance with human skin because of the superior skin adhesion. The acquired ECG signals possessed a higher signal-to-noise ratio compared with those recorded by other epidermal electrodes which have larger contact impedance with human skin. Figure 3g shows the ECG signals acquired by E-tattoo before fracture and after self-healing. It can be seen that whether E-tattoo was used before fracture or after self-healing, it is able to obtain almost the same high-quality ECG signals which can easily provide detailed information of each peak.

#### 3.1.2. EMG

**Figure 4:**
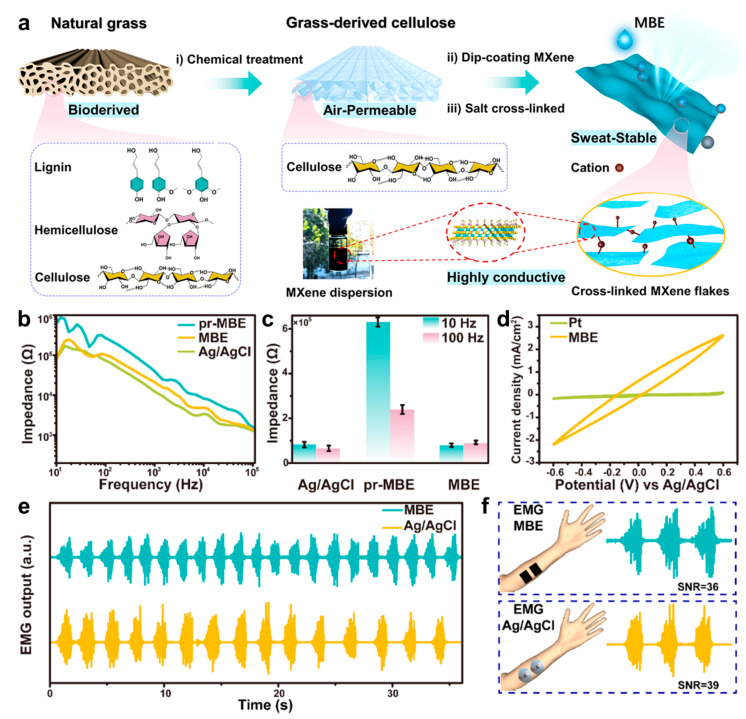
Fabrication procedures, performance characterization and application illustration of MXene-based electrodes (MBE). (**a**) Preparation process of MBE. (**b**) Contact impedances between skin and different electrodes, including pr-MBE, MBE and Ag/AgCl. (**c**) Comparison of impedance values between pr-MBE, MBE and Ag/AgCl electrodes at 10 and 100 Hz. (**d**) Cyclic voltammetry curves for Pt and MBE electrodes. (**e**) EMG signals recorded by Ag/AgCl and MBE electrodes. (**f**) Schematic illustration of MBE and Ag/AgCl electrodes attached on the arm for EMG measuring and the corresponding SNR values for two electrodes [123].

EMG is a common electrophysiological signal from muscle electrical activity, and can be used for determining the functional status of muscles, neurons and the neuromuscular junction [119,120,121]. In addition to these functions, EMG is also used to diagnose muscle diseases and trace recovery procedure in the muscle treatment process [122]. To guarantee validity for subsequent diagnoses, keeping accurate acquisition is of great significance for EMG. For this purpose, Song et al. designed a MXene-based epidermal electrode (MBE) using MXene sheets and a porous cellulose skeleton via a dip-coating method [123]. Figure 4a schematically illustrates the preparation process of MBE. First, cellulose film with porous structure was obtained after removing lignin and hemicellulose chemically from natural grass by NaClO_2_ and NaOH. The porous structure endows the MBE with good breathability, which facilitates sweat permeability and improves skin comfort when used as an epidermal electrode. To realize conductivity of the cellulose film, dip-coating was conducted to integrate MXene sheets with cellulose via van der Waals forces. The obtained conductive MBE was then cross-linked by soaking in artificial sweat to maintain conductivity and electrical stability in a wet environment. The contact impedances between skin and un-crosslinked MBE (pr-MBE), MBE and Ag/AgCl electrodes were measured, respectively, as presented in Figure 4b. Additionally, Figure 4c shows their contact impedances at frequencies of 10 and 100 Hz. It can be seen that pr-MBE exhibited much lower interfacial interaction with skin in comparison to MBE and Ag/AgCl electrodes, indicating that with sweat crosslinking with MXene, MBE’s impedance with skin was largely reduced. The reason of the lower impedance upon sweating was further investigated via cyclic voltammetry curves (Figure 4d), which mainly ascribed to increase effective surface area with MBEs due to the moisture wicking effect of cellulose. The MBE and Ag/AgCl electrodes were attached on the forearm for acquiring EMG signals, respectively, as shown in Figure 4e,f. The signal-to-noise ratios acquired by MBEs and Ag/AgCl electrodes were comparable, which were 36 dB and 39 dB, respectively. In addition to EMG signals acquisition, MBE also can be used for subsequent muscle disease treatment through electrical stimulation and electrothermal treatment. Therefore, the MBE with multiple functions of EMG diagnosis and muscle disease treatment is a kind of promising biomaterial-based epidermal electronics for electrophysiological acquisition and medical care.

#### 3.1.3. EEG

**Figure 5:**
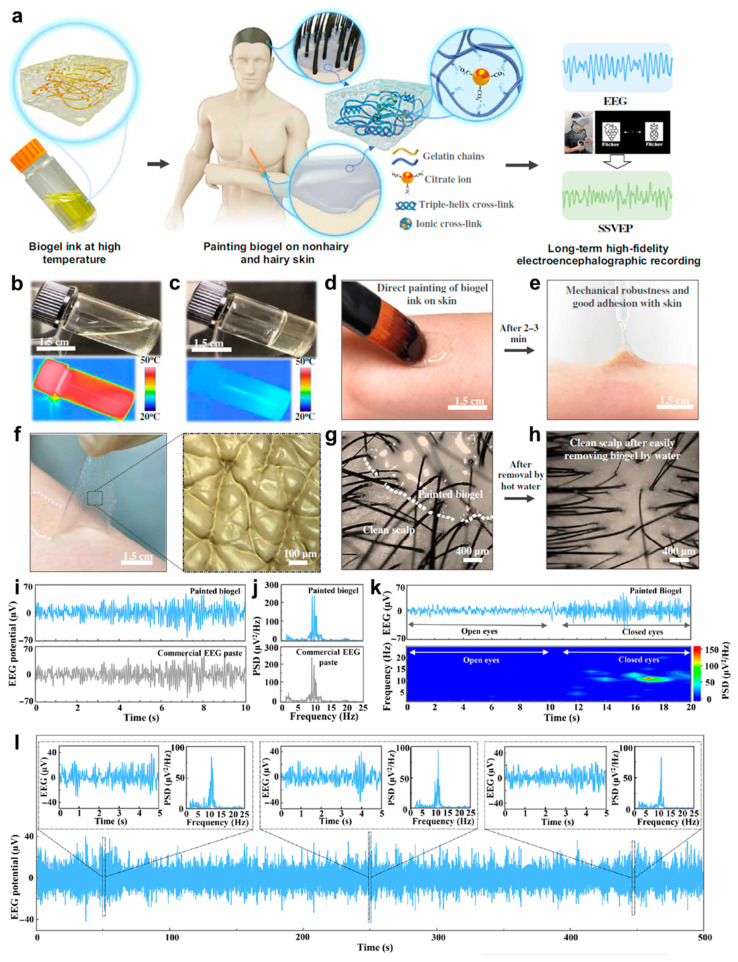
Concept illustration, performances characterization and application of gelatin-based biogel. (**a**) Conceptual graph of paintable biogel on hairy scalp for EEG monitoring. (**b**) Photograph of liquid biogel at high temperature. (**c**) Photograph of solid biogel at room temperature. (**d**) Photograph showing liquid biogel painted on skin directly. (**e**) Photograph of gelation and adhesion on skin for biogel at room temperature after 2–3 min. (**f**) Photograph (left) and optical image (right) of biogel peeled off from skin. (**g**) Optical image of hairy scalp coated by biogel. (**h**) Optical image of hairy scalp after removing biogel by hot water. (**i**) EEG signals acquired by commercial EEG paste (bottom) and gelatin-based biogel (top). (**j**) PSDA for EEG signals from (**i**). (**k**) EEG signals and corresponding spectrogram under opening eyes/closing eyes. (**l**) 500 s of EEG signals recorded by gelatin-based biogel. Insets illustrates partially enlarged images and the corresponding PSDA [131].

EEG, another important electrophysiological signal that reflects the electrical activity of the brain, is of great significance for the diagnosis of epilepsy, mental disorder and some brain diseases [124,125,126]. Generally, it is more difficult to obtain high-fidelity EEG signal due to poor mutual contact between electrodes and the hairy scalp [127]. In addition, the EEG signal is relatively weak compared with other physiological signals [128]. Traditional liquid EEG electrodes may cause leakage to be short-circuited, while solid electrodes usually have lower contact reliability [129,130]. Therefore, it is highly desirable to develop a comprehensive electrode that provides both good contact and stability. Taking advantage of the merits of gelatin, Wang et al. reported a biocompatible biogel with easy phase change ability between the liquid and solid [131]. Figure 5a illustrates that the gelatin-based liquid-state biogel could easily be painted on both non-hairy human skin and hairy scalp. After it was transformed to a solid state at lower temperature, EEG and steady-state visually evoked potentials (SSVEPs) could be acquired and classified at a long term. Figure 5b,c show that the biogel is in a fluidic state at high temperature and will transform to a solid phase when the temperature is dropped to room temperature due to the reversible noncovalent cross-links. Since it is totally based on biocompatible biomaterial, the liquid gel at high temperature could be directly painted on skin without any stimulation to human skin (Figure 5d). After 2–3 min at room temperature, it turned to a solid state which gained both superior mechanical robustness and favorable adhesion with skin (Figure 5e). Figure 5f further demonstrates the conformal contact between solid state biogel and the corrugated skin surface. Figure 5g clearly shows the painted gelatin-based biogel on the hairy scalp. After being treated with hot water, the solid state biogel changed to a fluidic state and could easily be cleaned up (Figure 5h). Then, the biogel was used as an epidermal electrode for recording the EEG alpha rhythm, as shown in Figure 5i. It can be seen that its fidelity was comparable to that of signals acquired by the commercial EEG electrode. In addition, the power spectral density analyses (PSDA) of the two electrodes were presented and compared in Figure 5j, which also exhibited no obvious difference. Figure 5k shows the EEG signals acquired by the biogel under eyes-open and eyes-closed states. Different from the eyes-open state, obvious alpha rhythm signals were observed in the eyes-closed state, which is the same as that of signals obtained by commercial EEG paste. Figure 5l further presents the long-term recording stability of the biogel for EEG recording. It can be seen that the obtained EEG alpha rhythm could last for 500 s, and characteristic peaks of alpha rhythm from PSDA at the beginning, middle and end of signals exhibited no clear difference. Ultimately, the biogel was used to capture SSVEPs for further application in virtual reality (VR), demonstrating the possibility of the brain–machine interface (BMI)-VR system.

In conclusion, due to various special properties of biomaterials, biomaterials-based epidermal electronics have potential in electrophysiological monitoring and play a similar role compared with traditional epidermal electronics. To further show the application of biomaterials-based epidermal electronics in ECG, EMG and EEG recording, the different biomaterials used for electrophysiological acquisition and corresponding parameters are summarized in Table 1.

### 3.2. Human–Machine Interactions

Biomaterials-based epidermal electronics also have great application prospects in human–machine interactions (HMIs) due to their versatile electrical functions, which can bring great convenience and entertainment to human society [142,143,144]. Via electrophysiological signals or human motion signals acquired by biomaterials epidermal electronics, HMIs including robot control, personal appliance control and virtual reality (VR) can be realized [145,146,147]. In this section, the general HMIs applications based on biomaterials-based epidermal electronics are discussed.

#### 3.2.1. Robot Control

**Figure 6:**
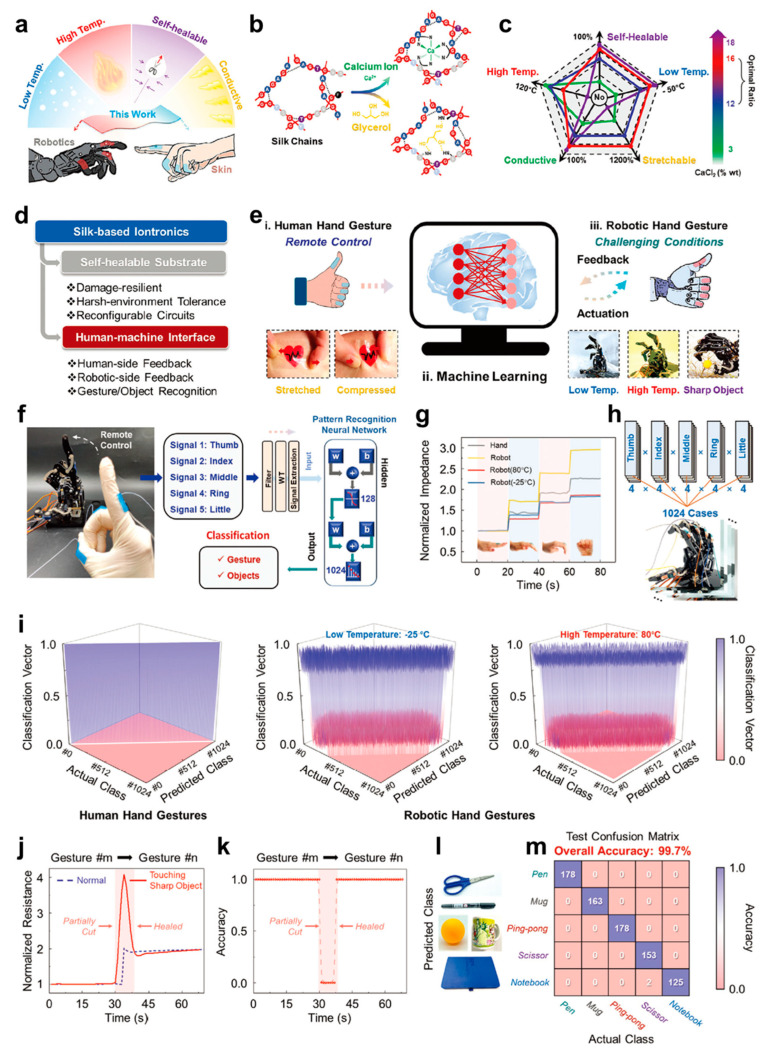
Performance and application of multifunctional silk protein-based iontronics. (**a**) Schematic illustration of the overall performances of iontronics. (**b**) Schematic illustration demonstrating metal coordination between Ca^2+^ and silk chains and hydrogen bonds between glycerol and silk chains. (**c**) Influence of Ca^2+^ content on various properties of silk-based iontronics. (**d**) The main features and application fields of silk-based iontronics. (**e**) Concept illustration of HMI system based on silk-based iontronics. (**f**) Photograph (left) of remote control for robotic hand by human hand and corresponding system (right) for classification of gesture and objects. (**g**) Normalized impedance for 4 gestures from human hand and robotic hand at high (80 °C and low (−25 °C) temperatures. (**h**) Signals from human and robotic hands for 1024 kinds of gestures. (**i**) Classification accuracy for human hand gestures and robotic hand gestures at −25 °C and 80 °C. (**j**) Normalized resistance changing with different gestures at normal state and partially cut state. (**k**) Recognition accuracy variation after being partially cut and healed. (**l**) Photographs of 5 objects with different shapes. (**m**) Test confusion matrix for recognition of different objects [150].

Traditional epidermal electronics that can accomplish HMI in human side signal collection are usually not suitable for utilization in the robot side which needs to maintain recognition capability in harsh environments [148,149]. To resolve this problem, Liu et al. developed eco-friendly silk protein-based iontronics by introducing the thermostability and freezing resistance of glycerol, providing the possibility for robot side application in harsh environments [150]. Figure 6a shows various characteristics of silk protein-based film, including frost-resisting, heat-resisting, self-healing, stretchability and conductivity. These features have endowed the film with good potential for both human side and robot side applications. Figure 6b illustrates the mechanism behind the silk protein’s various properties, in which metal coordination and hydrogen bonds were formed in silk protein due to the existence of Ca^2+^ and glycerol. Through adjusting the proportion of Ca^2+^, the comprehensive performance for iontronics could be optimized with an optimal ratio of 12–16 wt% for Ca^2+^ (Figure 6c). Figure 6d shows that the resulting iontronics can be utilized not only as a self-healable substrate, but also for constructing the HMI system. Assisting by machine learning technique, human fingers with the silk-based film adhering to them can control remotely the corresponding gestures of the robot, even in harsh environments (Figure 6e). Figure 6f specifically illustrates the process flow of robot control. After the signals were collected from human and robotic hand joints by silk-based film, the signals were processed and classified accurately by artificial neural network (ANN). Additionally, based on the above process of the iontronics, various bending degrees of joints in both human and robot can be sensed accurately, despite being in extreme environments (Figure 6g). Since four bending degrees for each finger were confirmed, accordingly, 1024 total gestures could be obtained (Figure 6h). Figure 6i demonstrates that besides the high accuracy in human gesture recognition, robotic gestures were also classified with high accuracy, despite being in extreme high temperature or low temperature owing to iontronics’ stable conductivity. To show the superiority of self-healing, the silk-based epidermal iontronics film was partially cut; within 15 s the function of the film was recovered for gesture recognition, and no negative influence on recognition accuracy was observed (Figure 6j,k). The gesture identifying accuracy for silk protein-based film was further investigated through grasping various objects with different sizes and shapes, as shown in Figure 6l,m. Scissor, mug, pen, notebook and ping-pong ball were successfully identified, respectively, and their accuracy can reach up to 99.7%. In general, the silk protein-based epidermal iontronics with good self-healing, decent stretchability, stable conductivity and harsh environment tolerance could promote the development of human–robotic interactions.

#### 3.2.2. Personal Device Control

**Figure 7:**
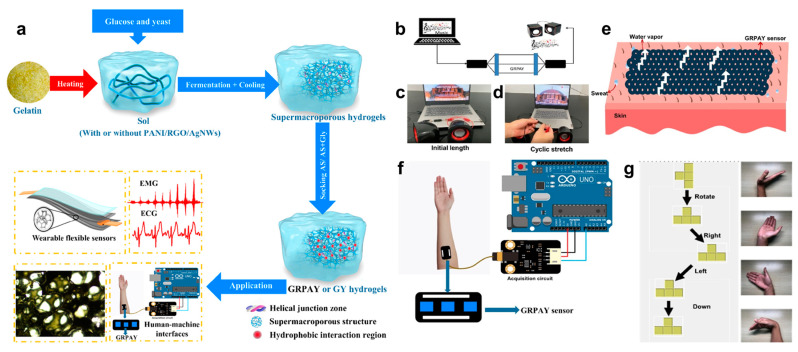
Preparation process and applications of GRPAY/GY hydrogels. (**a**) Fabrication procedure and corresponding applications of GRPAY/GY hydrogels. (**b**–**d**) Schematic illustration of GRPAY hydrogels cable for signal transmission, and photographs showing normal working state of GRPAY hydrogels cable under cyclic stretching. (**e**) Schematic diagram presenting breathability of the GRPAY sensor on skin. (**f**) HMI realized by GRPAY sensor. (**g**) Photographs illustrating playing Tetris game controlled by signals of 4 different gestures acquired from GRPAY sensor [135].

In addition to the robotic control, the control on personal devices is also a common application direction that brings convenience to daily life [151,152,153]. Inspired by fermentation, Cheng et al. proposed a gelatin-based hydrogel which has the controllable supermacroporous structure [135]. Figure 7a shows the detailed fabrication process of the hydrogel. First, plate count agar (PCA) solution was mixed with gelatin obtained from nature; the mixture was then blended with glucose and yeast solution. In the process, temperature was adjusted continually for activating fermentation to obtain a GY hydrogel. Subsequently, RGO/polyaniline (PANI)/AgNWs with stable conductivity were introduced into GY solution to obtain GRPAY hydrogels. For the purpose of enhancing water retention ability, the fabricated two hydrogels were soaked in ammonium sulfate (AS)/AS-glycerin to get ultimate GY or GRPAY hydrogels. As a wearable flexible epidermal sensor, GRPAY hydrogel with even supermacroporous structure can be used for detecting EMG and ECG signals, and the acquired EMG signals were further used for HMI applications. First, the GRPAY hydrogel was used as cables to connect a loudspeaker and a computer, as shown in Figure 7b–d. It is observed that the music signal from the computer was transmitted to the loudspeaker by GRPAY cable even when it was repeatedly stretched, indicating its good stretchability and electrical stability. Because of the supermacroporous structure and biocompatibility, GRPAY hydrogel was then adhered on skin without any stimulation, and sweat vapor can also be permeable, which proved good wearable comfort of the GRPAY sensor (Figure 7e). Therefore, the gelatin-based epidermal sensors with stable conductivity and high sensitivity were attached on the human arm and successfully applied in the HMI system, as shown in Figure 7f. Figure 7g shows that four different gestures corresponding to the commands of right, left, rotation and down were designed to play the Tetris game. Then, the signals of four gestures collected by GRPAY hydrogel were defined as various game actions to achieve corresponding functions. In summary, owing to the biocompatibility, environmental tolerance and stretchability, the natural biomaterials-based epidermal electronics can provide an ideal path to drive the progress of HMI.

#### 3.2.3. Virtual Reality

**Figure 8:**
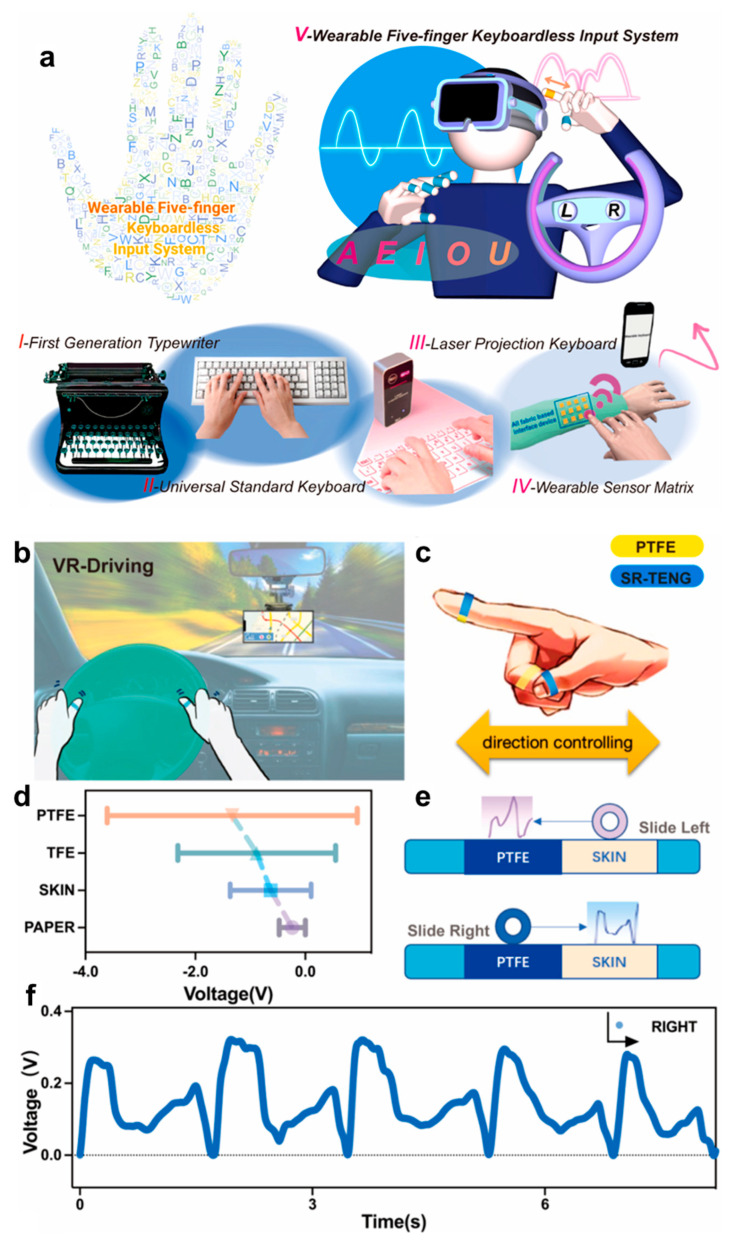
Concept and application of SF ring TENG (SR-TENG) in wearable keyboardless input system. (**a**) The development process of keyboard. (**b**) Schematic illustration for application of SR-TENG in VR driving. (**c**) Direction controlling realized by SR-TENG during finger sliding. (**d**) Voltage changing range with different materials contacting with SR-TENG. (**e**) Schematic diagram of direction controlling by SR-TENG. (**f**) Voltage variation produced by SR-TENG during sliding to right [158].

VR is an emerging technology that can directly exert the feeling of visual, tactile and auditory senses on users, which has attracted increasing attention in industrial engineering, entertainment, medical treatment, etc. [154,155,156]. Biomaterials-based epidermal sensors that can convert external stimuli into electrical signals have great application prospects in VR due to their excellent biocompatibility, sensitivity and wearability [131,157]. Liu et al. developed a silk fibroin (SF) based triboelectric nanogenerator (TENG) [158]. Combining with a number of pair encoding tables, a wearable five-finger keyboardless input system (WKIS) was constructed for application in VR-driving games. Figure 8a illustrates the development stages of the keyboard, in which the keyboards changed from first generation typewriter to wearable sensor matrix. However, they all have matrix keyboards with the traditional keyboard design, which has limited functions. Therefore, the wearable five-finger keyboardless input system developed a new input method with no need for a matrix keyboard. SF and silver nanowires coated between two SF films were made into rings to obtain a SF ring TENG (SR-TENG). When fingers are performing motions, corresponding electrical signals will be generated with SR-TENG on the finger contact and will separate the reference electrode, which is consistent with the working principle of typical nanogenerators [159,160]. Hence, finger motions were converted into electrical signals. The direction identification for finger motions can also be achieved owing to the different voltage variation when skin or PTFE contacted SR-TENG (Figure 8c,d). Figure 8e illustrates the process and electrical signals of the SR-TENG on the finger that slides left from skin to PTFE or slides right from PTFE to skin. It can be seen that the motion of sliding left and right can be distinguished by the different signals. Figure 8f demonstrates the voltage variations of SR-TENG when it came in contact with PTFE then slipped to skin. Based on these properties, the SR-TENG can be applied in VR games or be used as a steering wheel in VR driving (Figure 8b). In addition, a new input method was exploited, which was realized by coding different signals acquired by five fingers. Specifically, the signals from thumb to little finger were separately coded as 1, 2, 3, 4, 5, and different letters will be displayed if the volunteer tapped his fingers twice in one second. For example, the alphabet “K”, which corresponds to code (3,2), can be output through clicking the middle finger and index finger in one second. In conclusion, the developed SR-TENG holds great potential to be applied on human skin for security defense and VR applications.

## 4. Conclusions and Future Perspectives

In conclusion, various types of biomaterials such as proteins and polysaccharides can be utilized in epidermal electronics. With multiple unique merits of biodegradability, biocompatibility, low cost and natural abundance, biomaterials are considered as ideal substitute materials for synthetic polymers to construct epidermal electronics, in order to resolve the problems of e-waste and skin safety issues. In addition, by integrating with diverse functional materials such as ions, CNTs, metals, or other synthetic polymers, biomaterials-based epidermal electronics are endowed with many additional functions, for example good conductivity, anti-freezing, heat-resisting and self-healing. Meanwhile, by unique structure design, epidermal electronics can possess more special properties, including gas permeability and superior stretchability. With these unique merits, the biomaterials-based epidermal electronics can be broadly applied in various applications including electrophysiological monitoring and HMI. Although natural biomaterials have many advantages over synthetic materials when used for epidermal electronics, there are still some issues that need to be resolved.

Firstly, to achieve some specific properties such as high conductivity and self-healing, some additional synthetic materials are needed to be mixed with biomaterials, thus deteriorating the biodegradability and biocompatibility of the epidermal electronics. Therefore, it is desirable to develop a fully biomaterials-based epidermal electronics that can realize some particular functions without the addition of other synthetic functional materials. In addition to materials, biomaterials-based epidermal electronics are still in the primary research stage, and usually have relatively poor performances that are not comparable to commercial epidermal devices. Accordingly, it is urgent to keep improving the performance of biomaterials-based epidermal electronics by using low cost and green production technology. Finally, due to relative inferior performances compared with traditional epidermal electronics, a limited application range for biomaterials-based epidermal electronics is also a crucial problem. In general, there still exist many challenges for fully bio-friendly and eco-friendly biomaterials, but they undoubtedly demonstrate great promise for the next-generation sustainable and biocompatible high-performance epidermal electronics.

## Figures and Tables

**Table 1:** Biomaterials used in electrophysiological recording and corresponding parameters.

Physiological Signal	Functional Materials	Included Biomaterials	Signal to Noise Ratio (SNR)	Interfacial Impedance	Ref.
ECG	Ca^2+^-modified silk	Silk fibroin	n/a	1.5 kΩ (10^6^ Hz)	[132]
ECG	PEDOT:PSS/glycerol-plasticized porous silk fiber	Silk	n/a	~5 kΩ (10^3^ Hz)	[133]
ECG	Ppy@AM-SF/CNC electrode	Silk fibroin	n/a	~1 kΩ (10^3^ Hz)	[134]
ECG	RGO/Gelatin/AgNWs	Gelatin	n/a	~15 kΩ (10^2^ Hz)	[135]
EMG	MXene/Cellulose electrode	Cellulose	36 dB	~1 kΩ (10^5^ Hz)	[123]
EMG	SiO_2_/carbon nanofibrils	Cellulose	28 dB	n/a	[136]
EMG	Hexamethylene diisocyanate cross-linked sericin-graphene textile	Silk sericin	n/a	~2 kΩ (10^4^ Hz)	[137]
EMG	Silk/Ca^2+^/FA/Au	Silk fibroin	n/a	~1 kΩ (10^4^ Hz)	[138]
EEG	Gelatin/Citrate ion	Gelatin	n/a	6.95 kΩ (10^3^ Hz)	[131]
EEG	Mesoporous cellulose membrane/NaCl	Cellulose membrane	n/a	6.64 kΩ(10^3^ Hz)	[139]
EEG	Chitosan/Au-TiO_2_	Chitosan	n/a	5 kΩ (10^3^ Hz)	[140]
EEG	Single-wall carbon nanotube/gelatin	Gelatin	14.81 dB	1.12 kΩ (10^5^ Hz)	[141]

## Data Availability

Data sharing not applicable.
